# Ferulsinaic Acid Modulates SOD, GSH, and Antioxidant Enzymes in Diabetic Kidney

**DOI:** 10.1155/2012/580104

**Published:** 2012-09-06

**Authors:** Ahmed Amir Radwan Sayed

**Affiliations:** ^1^Biochemistry Department, Faculty of Science, King Abdulaziz University, P.O. Box 80203, Jeddah 21589, Saudi Arabia; ^2^Chemistry Department, Faculty of Science, Minia University, El-Minia 61519, Egypt

## Abstract

The efficacy of Ferulsinaic acid (FA) to modulate the antioxidant enzymes and to reduce oxidative stress induced-diabetic nephropathy (DN) was studied. Rats were fed diets enriched with sucrose (50%, wt/wt), lard (30%, wt/wt), and cholesterol (2.5%, wt/wt) for 8 weeks to induce insulin resistance. After a DN model was induced by streptozotocin; 5, 50 and 500 mg/kg of FA were administrated by oral intragastric intubation for 12 weeks. In FA-treated diabetic rats, glucose, kidney/body weight ratio, creatinine, BUN, albuminurea, and creatinine clearance were significantly decreased compared with non treated diabetic rats. Diabetic rats showed decreased activities of SOD and GSH; increased concentrations of malondialdehyde and IL-6 in the serum and kidney, and increased levels of 8-hydroxy-2′-deoxyguanosine in urine and renal cortex. FA-treatment restored the altered parameters in a dose-dependent manner. The ultra morphologic abnormalities in the kidney of diabetic rats were markedly ameliorated by FA treatment. Furthermore, FA acid was found to attenuate chronic inflammation induced by both Carrageenan and dextran in rats. We conclude that FA confers protection against injuries in the kidneys of diabetic rats by increasing activities of antioxidant enzymes and inhibiting accumulation of oxidized DNA in the kidney, suggesting a potential drug for the prevention and therapy of DN.

## 1. Introduction

Diabetes mellitus (DM) is a life-threatening metabolic disorder and the disease is becoming a serious social problem. Hyperglycemia is the major cause of diabetic complications, such as retinopathy, nephropathy, and neuropathy [1, 2].

Diabetic nephropathy (DN) is characterized by structural abnormalities including hypertrophy of both glomerular and tubular elements, increase in the thickness of glomerular basement membranes, and progressive accumulation of extracellular matrix components [3]. It also results in functional alterations including the early increase in the glomerular filtration rate with intraglomerular hypertension, subsequent proteinuria, systemic hypertension, and eventual loss of renal function [3]. The development of irreversible renal change in diabetes mellitus such as glomerulosclerosis and tubulointerstitial fibrosis results ultimately in end stage renal disease [1]. Although adequate control of blood glucose levels may prevent the development of complications, it is difficult to achieve strict blood glucose control, leading to a year-by-year increase in the number of patients with diabetes [4]. 

Although the mechanism of DN has not yet been clarified because of the complexity of the pathophysiology of DM, numerous factors have been reported to be involved, including the activation of the renin—angiotensin system [5], activation of protein kinase C*β* [6], activation of nuclear factor kaba B (NF-*κ*B) [7], enhanced formation of advanced glycation end products (AGEs) [8], and acceleration of oxidative stress [9]. Many experimental evidences suggest the involvement of free radicals in the pathogenesis of diabetes [10] and more importantly in the development of diabetic complications [11]. Free radicals are capable of damaging cellular molecules, DNA, proteins, and lipids leading to altered cellular functions. Many recent studies reveal that antioxidants capable of neutralizing free radicals are effective in preventing experimentally induced diabetes in animal models [12, 13] as well as reducing the severity of diabetic complications [11]. Ferulic acid, an antioxidant of plant cell wall, was reported to prevent functional and pathological abnormalities in the kidney of diabetic rats reducing oxidative stress and inflammation [14, 15].

**Scheme 1:**
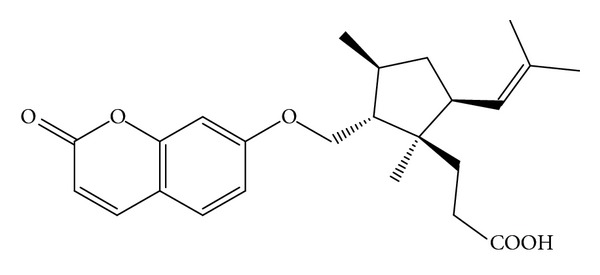


Many plants synthesize an array of chemical compounds that are not involved in their primary metabolism. These “secondary compounds” instead of serving a variety of ecological functions, they ultimately enhance the plant's survival during stress. In addition, these compounds may be responsible for the beneficial effects of fruits, vegetables, and many plants on an array of health-related measures. Traditional herbal medicines have been employed for thousands of years and have contributed greatly to the prevention and treatment of various diseases, including diabetes. They are still valuable for human health and have received much attention as potential sources of new therapeutic agents due to their varied biological activity and low toxicity [16]. *Ferula sinaica L*. (Apiaceae) has some 130 species distributed throughout the Mediterranean area and central Asia. These plants are used in Egypt as spices and in the preparation of local drugs. The resins are reported to be used for stomach disorders such as a febrifuge and carminative agent and in the treatment of skin infections and hysteria [17]. Previous work showed that the main constituents of this genus are sesquiterpenes and sesquiterpene coumarins [18]. Ferulsinaic acid (FA) is the first member of a new rearranged class of sesquiterpene coumarins from the genus *Ferula*. It was isolated from *F. sinaica L*. The molecular formula of FA is found to be C_24_H_30_O_5_. The structure of FA was established in a previous work of our research group [19] as indicated below in Scheme 1.

Extracts of *F. sinaica* was found to inhibit the spontaneous movements of rabbit jejunum and guinea pig ileum and acetylcholine induced contractions. Extracts also inhibited the contractions of rabbit tracheal smooth muscle induced by acetylcholine stimulation and the contractions of guinea pig tracheal smooth muscle induced by histamine stimulation. In addition, the extract inhibited the contractions of rabbit aorta induced by norepinephrine stimulation [20]. Furthermore, extracts of *F. sinaica* inhibited the spontaneous movements of rat and guinea pig uterine smooth muscle and also the contractions induced by oxytocin stimulation and have some antioxytocic potential [21].

FA was found to extend life span of wildtype *Caenorhabditis elegans (C. elegans)* under standard condition. Moreover, resistance to both heat stress and induced chemical stress of *C. elegans* were improved. Furthermore, FA was found to attenuate the formation reactive oxygen species (ROS) and advanced glycation end products (AGEs) in *C. elegans* [22].

In the present study, the antioxidant power as well as the anti-inflammatory effect of FA were examined to evaluate its efficacy to attenuate ROS production, inflammation, and to modulate the antioxidant enzymes of diabetic kidney in rats. 

## 2. Materials and Methods

### 2.1. Animals

Adult male Albino rats weighing 180 to 200 g were used throughout this study. The animals were housed in cages and received normal rat chow and tap water *ad libitum* in a constant environment (room temperature 28 ± 2°C, room humidity 60 ± 5%) with a 12 h light and 12 h dark cycle. The animals were kept under observation for one week prior to the start of the experiments. 

### 2.2. Isolation and Purification of FA

Air-dried roots of *F*. *sinaica* were collected from North Sinai Peninsula, El-Arish, Egypt. 15 kg of *F*. *sinaica* were ground and extracted with CH_2_Cl_2_ at room temperature. The extract was concentrated to obtain a residue of 1100 g. The residue was fractionated by silica gel CC (6 · 120 cm) eluted with hexane, followed by gradient elution with hexane–CH_2_Cl_2_ up to 100% CH_2_Cl_2_ and finally with CH_2_Cl_2_–MeOH (85 : 15). The hexane–CH_2_Cl_2_ extract (1 : 3, 140 g) was purified by HPLC (MeOH–H_2_O, 73 : 27) to afford FA (100 mg) [19].

### 2.3. Induction of DN Model and Study Design

Seventy rats were used in this experiment. Rats were fed diets enriched with sucrose (50%, wt/wt), lard (30%, wt/wt), and cholesterol (2.5%, wt/wt) for 8 weeks to induce insulin resistance. Ten rats were used as control group (group 1, *n* = 10), which received a single tail vein injection of 0.1 mol/L citrate buffer only. A group of 60 rats were intravenously injected with STZ (65 mg/kg body weight), which was freshly prepared in a 0.1 mol/L citrate buffer (pH 4.5), after fasting for 12 hours. Only rats with blood glucose higher than 200 mg/dL after 5 days will be considered as being diabetic in the fasting state, by using One Touch *select* analyzer (Life Scan, Inc., UK). Rats with blood glucose lower than 200 mg/dL were excluded from the study. All studies were carried out one week after STZ had been injected. Fifty diabetic rats were randomly divided into 5 groups: DN, diabetic and treated with metformin-HCl (MF) (125 mg kg^−1^ d^−1^; DN + MF) [23], diabetic and treated with a low dose (5 ng kg^−1^ d^−1^) of FA (DN + FA1), diabetic and treated with a medium dose (50 ng kg^−1^ d^−1^) of FA (DN + FA2), and diabetic and treated with a high dose (500 ng kg^−1^ d^−1^) of FA (DN + FA3). The LD_50_ of FA was found to be 2 *μ*g/kg. The MF and FA were administered with distilled water via intragastric intubation. Treatments were continued for 12 weeks. Body weight and blood glucose levels were measured regularly. At the end of the experiment, animals were sacrificed using ether anaesthesia. Kidneys were dissected and rinsed with cold PBS and then weighed. An index of renal hypertrophy was estimated by comparing the wet weight of the left kidney to the body weight.

### 2.4. Kidney Homogenate Preparation

Every kidney tissue was cut into small pieces and washed by phosphate-buffered saline. Furthermore, it was grinded in a homogenization buffer {0.05 M Tris-HCl pH 7.9, 25% glycerol, 0.1 mM EDTA, and 0.32 M (NH_4_)_2_SO_4_} containing a protease inhibitor tablet (Roche, Germany). The lysates were homogenized on ice using a Polytron homogenizer. The solution has been sonicated in an ice bath to prevent overheating for 15 seconds followed by centrifugation at 12000 rpm, 4°C for 5 minutes. The supernatant was aliquoted and stored at −80°C and assayed for protein concentration using BCA kit (Pierce, Rockford, USA) using albumin diluted in a lysis buffer as a standard. The homogenate was used for the determination of reduced glutathione (GSH), level of lipid peroxidation (MDA), concentration of *N*ε**-carboxymethyl lysine (CML), activity of superoxide dismutase (SOD), and level of IL-6. The other kidneys from each group were used for histopathological examination, determination of the level of AGEs, and for isolation of renal DNA.

### 2.5. Blood Sampling and Analysis

Blood samples of rats were centrifuged at 2,000 g for 10 minutes at 4°C, and aliquoted for the respective analytical determinations. The diagnostic kits for determinations for plasma levels of glucose, creatinine (Cr), blood urea nitrogen (BUN), sodium, and potassium were purchased from BioSystem (Barcelona, Spain). All analyses were performed in accordance with the manuals provided by the manufacturer.

### 2.6. Analysis of Urine Parameters

Urine samples were collected by placing the rats in individual metabolic cages for 24 h before diabetes had been induced and the day before the end of treatment. The urine albumin concentration was determined with an ELISA kit (Nephrat II, Exocell, Philadelphia, PA, USA) and the concentration of Cr in pooled urine samples was determined by the commercial assay kit. All analyses were performed in accordance with the manuals provided by the manufacturers. The 24 h urinary albumin excretion rate (UAER) was calculated as UAER (**μ**g 24 h*−*1) = urinary albumin (**μ**g mL^−1^) × 24 h urine volume (mL). Cr clearance (Ccr) was calculated using the following equation: Ccr (mL min^−1^ kg^−1^) = (urinary Cr (mg dL^−1^) × urinary volume (mL)/serum Cr (mg dL^−1^)) × (1000/body weight (g)) × (1/1440 (min)) [24]. 

### 2.7. Determining Enzymatic Activities

The activities of total SOD (EC: 1.15.1.1) as well as the concentrations of MDA and GSH in the kidney homogenate were determined using commercially available kits from BioVision Research Products (Linda Vista Avenue, USA) according to the methods described by Nishikimi et al., and Sayed [25, 26]; Ohkawa et al. and Mekheimer et al. [27, 28], and Moron et al. [29], respectively.

### 2.8. Determination of IL-6

IL-6 concentration in the serum and in the kidney homogenate was determined by an ELISA kit. The ELISA for determination of IL-6 was performed using a commercially available kit from R&D (Mannheim, Germany) according to the instructions of the manufacturer. 

### 2.9. Measurement of Urinary and Renal 8-Hydroxy-2′-deoxyguanosine

Urinary 8-hydroxy-2′-deoxyguanosine (8-OHdG) levels were determined using an enzyme-linked immunosorbent assay kit from Genox Corporation (Baltimore, MD. USA) according to the method of Matsubasa et al. [30] and corrected by using individual urine creatinine concentrations. Extraction of renal DNA was performed using a DNA extraction kit (Promega, Germany) following the manufacturer's protocol. The genomic DNA samples from kidney tissue were also used for the determination of 8-OHdG using the competitive ELISA kit.

### 2.10. Renal AGEs Level

The renal AGE level was determined according to a previous method with slight modifications [31, 32]. Minced kidney tissue was delipidated with chloroform and methanol (2 : 1, v/v) overnight. After washing, the tissue was homogenized in 0.1N NaOH, followed by centrifugation at 8,000 g for 15 min at 4°C. The amounts of AGEs in these alkali-soluble samples were determined by measuring the fluorescence at an emission wavelength of 440 nm and an excitation wavelength of 370 nm using a fluorescence spectrophotometer (Hitachi, Japan). A native bovine serum albumin (BSA) preparation (1 mg mL^−1^ of 0.1N NaOH) was used as a standard. The fluorescence values of samples were measured at a protein concentration of 1 mg mL^−1^ and expressed in AU compared with a native BSA preparation.

### 2.11. Assessment of Renal CML

The supernatant of the kidney homogenate was tested for CML using the anti-CML rat autoantibody ELISA kit which employs the semiquantitative enzyme immunoassay technique. The absorbance of the resulting yellow product is measured at 450 nm [33].

### 2.12. Histopathological Examination

Renal tissues were collected after animal sacrifice, fixed in 10% formalin, processed routinely, and embedded in paraffin. 5-*μ*m thick sections were prepared and stained with periodic acid Schiff (PAS). Glomerular histopathological changes and mesangial lesions were scored in term of the glomerular mesangial expansion (increase in the mesangial matrix) [34].

In order to evaluate the anti-inflammatory activity of FA on the acute inflammation, carrageenan-induced rat paw edema and dextran-induced rat paw edema were estimated as described by Arunachalam et al. [35] with slight modification. 

### 2.13. Carrageenan-Induced Rat Paw Edema

Thirty rats were divided into 5 groups, each 6 rats. FA at 5, 50, and 500 ng/kg and indomethacin at 10 mg/kg body weight in olive oil were given to rats orally 30 min before carrageenan injection. The same volume of the vehicle was given to control group. The left rear plantar region of the rats was injected with 0.1 mL of carrageenan (1% in saline). The edema produced was determined by measuring the difference of the paw diameter using an analogic pakimeter (vernier) before carrageenan injection and at 0, 3, and 5 h after carrageenan injection. 

### 2.14. Dextran-Induced Rat Paw Edema

The paw edema was induced in the right hind paw by subplantar injection of 0.1 mL of freshly prepared 1% dextran solution. Paw thickness was measured at 0, 45, and 90 min after dextran injection. The rats were treated as above. The percentage of inhibition was calculated.

### 2.15. Statistical Analysis

All group values are expressed as the mean ± SD. Data was evaluated using the Sigma Stat (version 13.0) statistical analysis program (by using SPSS 11.09 for windows). An analysis of variance test was performed initially to test differences in the treatment. After the analyses of variance, a Tukey post-hoc test was performed to examine whether there were any significant differences between different treatment groups, the level of significance was set at *P* < 0.05.

## 3. Results

### 3.1. Effects of FA Treatment on Blood Glucose and Kidney/Body Weight Ratio

Data in Table 1 showed that the STZ injection resulted in a nearly 5-fold increase of the fasting blood glucose levels in the Albino rats. At the end of the 12-week period, the final kidney/body weight ratios of untreated diabetic animals were significantly higher than those of control animals (*P* < 0.05). FA-treated diabetic animals showed a significant reduction of this kidney/body weight ratio, which approached the levels of the MF group compared with the diabetic animals (*P* < 0.05). Moreover, there was also a significant correlation (*P* < 0.05) between the dosage groups.

### 3.2. Effects of FA Treatment on Renal Function

Table 1 shows that the BUN, creatinine, 24-hour Upro, sodium, potassium, and Ccr levels were significantly higher in the DN group than in the normal control group (*P* < 0.05). The low dose of FA markedly reduced Upro, potassium, and Ccr in the DN group but did not reduce serum BUN and Scr. The medium and high doses of FA significantly reduced all previously listed renal functional parameters in the DN rats (*P* < 0.05).

### 3.3. Effects of FA Treatment on Activities of Antioxidant Enzymes and Oxidative Stress Markers

The activity of SOD and concentration of GSH were lower, whereas concentration of MDA was higher in the kidney homogenate of the DN group than the control group (*P* < 0.05), suggesting that these rats suffered from oxidative stress (Table 2). Treatment with medium and high doses of FA significantly decreased the concentrations of MDA and significantly increased SOD activity and GSH concentrations (*P* < 0.05). These results indicate that FA ameliorates oxidative stress in DN rats. Metformin HCl significantly increased the antioxidant enzymes activities (*P* < 0.05).

### 3.4. Effects of FA Treatment on Serum and Renal IL-6

Diabetes significantly increases the degree of inflammation and the release of IL-6 in DN group compared with the normal control group (*P* < 0.05). Treatment of animals with FA and metformin appreciably attenuated this inflammation and IL-6 production in all treated groups compared with DN group (*P* < 0.05), Tables 1 and 2.

### 3.5. Effects of FA on the Renal AGEs and CML

As a result of diabetes the renal levels of AGEs and CML were significantly elevated in the STZ-diabetic rats. These elevated levels were effectively lowered in a dose-dependent manner by FA treatment for 12 weeks (Table 2).

### 3.6. Effects of FA Treatment on Urinary and Renal 8-OHdG

**Figure 1:**
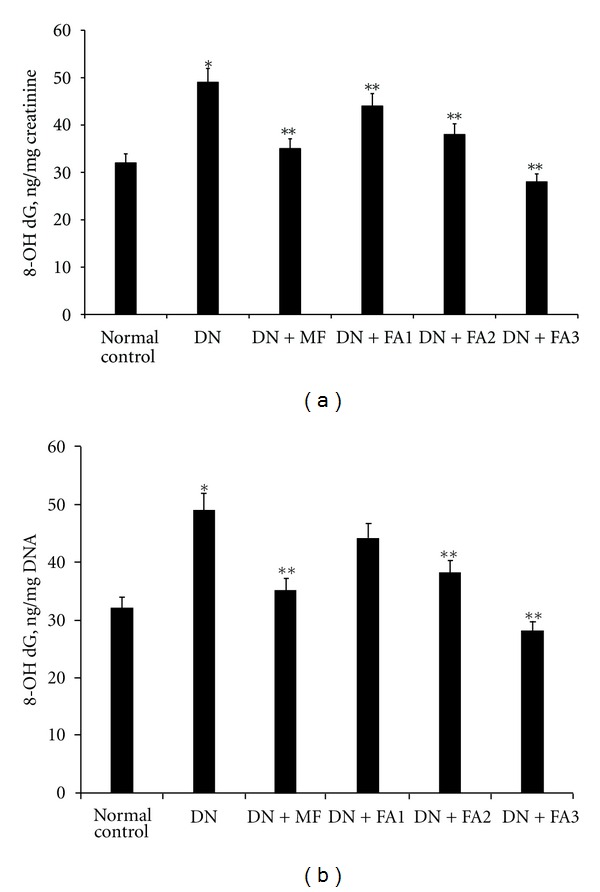
8-Hydroxy-2′-deoxyguanosine levels in the urine (a) and renal cortex (b) of rats. Urinary and renal cortex 8-OHdG levels in diabetic rats were significantly attenuated in a dose-dependent manner after FA administration. Data are expressed as the means ± SD. DN indicates diabetes untreated group; FA1, FA2, and FA3, diabetic rats treated with low dose (5 ng/kg) of FA (DN + FA1), middle dose (50 ng/kg) of FA (DN + FA2), and high dose (500 ng/kg) of FA (DN + FA3), respectively. *P < 0.05 versus normal control group, **P < 0.05 versus diabetes untreated group.

The total amounts of urinary 8-OHdG excretion were significantly greater in STZ-induced diabetic rats than in control rats at 12 weeks after the onset of diabetes (*P* < 0.05). Administration of FA suppressed the increase in urinary excretion of 8-OHdG in the diabetic rats to the same extent as MF (*P* < 0.05). In parallel with the urine results, the levels of 8-OHdG in the DNA were markedly increased in the kidney of diabetic rats, and those increases were reversed by FA treatment (*P* < 0.05). In addition, the magnitude of these increases was reduced by FA treatment in a dose-dependent manner (*P* < 0.05; Figures 1(a) and 1(b)).

### 3.7. Histopathological Findings

**Figure 2:**
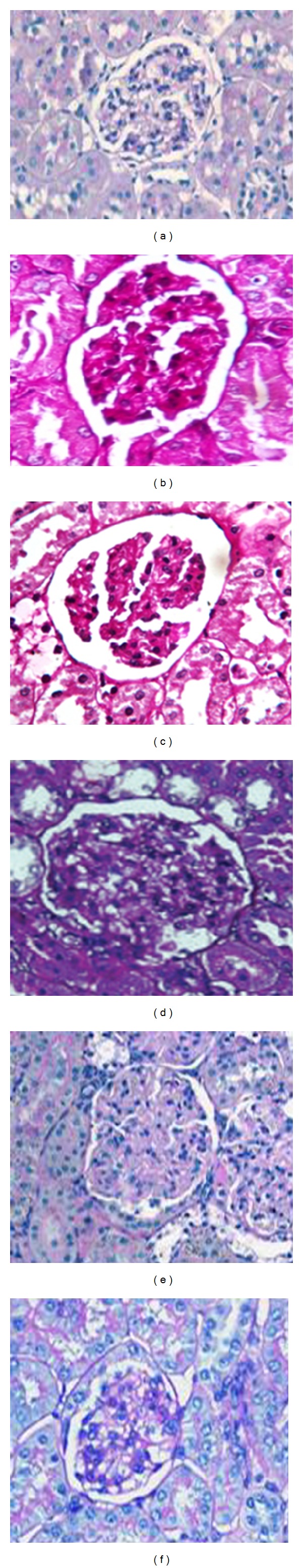
Figure (2): PAS staining of kidney sections of normal control rats (a), DN rats (b), DN treated with MF (c), FA1 (d), FA2 (e), and FA3 (f), respectively. Increased mesangial matrix, thickened CBMs, TBMs, and GBMs are present in the glomerulus of diabetic untreated rats as compared with the control and diabetic treated rats.

A significant enlargement of the glomeruli, thickening of capsular basement membranes (CBMs), glomerular basement membranes (GBMs), and tubular basement membranes (TBMs), increased amounts of mesangial matrix, and tubular dilatation were observed in diabetic untreated rats (Figure 2(b)). The renal histology in untreated diabetic rats showed accelerated mesangial expansion, thickening of CBMs, GBMs, and TBMs, characterized by an increase in PAS-positive area as compared with control animals (Figures 2(a) and 2(b)). The treatment of rats with FA and MF reduced the glomerular size, thickening of CBMs, GBMs, and TBMs, increased amounts of mesangial matrix, and tubular dilatation as compared with diabetic untreated rats (Figures 2(c)–2(f)).

### 3.8. Effect of FA on the Acute Inflammation

In carrageenan-induced inflammatory rat models, FA at concentrations 5, 50 and 500 ng/kg inhibited the edema formation in the third hour by 38% (*P* < 0.05), 42%, and 57% (*P* < 0.005) in a dose dependent manner, respectively. This effect also extended and significantly increased up to the fifth hour (*P* < 0.005). In addition, in dextran-induced inflammatory model, FA at 45 min inhibited the paw edema by 42%, 49%, and 57% (*P* < 0.005) of inhibition at the concentrations of 5, 50 and 500 ng/kg, respectively. At 90 min, there was an increase in the percentage of inhibition by 47%, 53% and 62%, respectively as shown in Tables 3(a) and 3(b)

## 4. Discussion

DN is one of the major microvascular complications of diabetes mellitus. Hyperglycemia can lead to both a rise in reactive oxygen species (ROS) production and the attenuation of free radical scavenging compounds [36]. In the present study, the development of DN was confirmed by significant elevations of kidney/body weight ratio, Scr, sodium, potassium, and BUN as well as Upro in diabetic rats, as earlier reported by other groups [14, 15, 23, 28, 37]. Oxidative stress can also affect nucleic acids resulting in modified DNA bases. 8-Hydroxy-2′-deoxyguanosine is a major product of DNA damage, and the measurement of its serum or urinary level provides information on various degrees of oxidative stress at the DNA level [38]. Urinary 8-OHdG excretion was significantly increased 12 weeks after the onset of diabetes. In addition, the renal cortex showed a markedly high expression of 8-OHdG in DNA. Moreover, we found for the first time that this oxidative damage is attenuated by FA treatment in a dose-dependent manner. Furthermore, based on the obtained data, urinary and renal 8-OHdG were significantly higher in the DN group than in the control group, which suggests that the observed renal injuries were attributed to high 8-OHdG levels in the diabetic kidney. However, FA administration prevented all the above functional and morphologic changes and maintained them near normal. The obtained data indicate that FA exerts its protective effects on the renal injury of diabetic rats via inhibiting the accumulation of oxidized DNA in the kidney.

Oxidative stress influences the pathogenesis of DN and is not only through overproduction of ROS but also through the reduction of antioxidant enzyme activities, the formation of lipid peroxides, and nonenzymatic protein glycosylation. Antioxidant enzymes are induced to protect against cellular and tissue injury. An imbalance between the production of ROS and antioxidants is believed to be involved in diabetes-induced renal failure [38]. Induction of diabetes in the present study caused a significant elevation of MDA and a reduction of both SOD activity and GSH concentration in the kidneys, as compared with the control group. Treatment with FA for 12 weeks reversed these oxidant/antioxidant parameters. The reversal of the oxidative damage due to FA is shown as a measure of antioxidant enzymes and indicates that it has possible antioxidant properties, which plays a crucial role in the defense against oxygen free radicals. The obtained data are in line with previous work [14, 15, 39].

The hyperglycemia condition results in irreversible tissue damage by the protein glycation reaction, which leads to the formations of glycosylated proteins and AGEs [40]. AGEs accumulation in the kidney have been regarded as an index of progressive renal damage in DN. CML is formed during the metal-catalyzed oxidation of polyunsaturated fatty acids in the presence of protein [41]. Therefore, CML could serve as a general biomarker of oxidative stress resulting from carbohydrate and lipid oxidation reactions. AGEs have the ability to activate NF-**κ**B signaling pathway which regulate the expression of many inflammatory genes like IL-6 [8, 42]. To test this hypothesis, we measured AGEs, CML, and IL-6 in the different groups. Actually, not only the overexpression of AGEs but also the higher levels of IL-6 in kidney of diabetic untreated rats were alleviated by 12 weeks of FA treatment. It seems that FA influenced not only the AGE-RAGE signaling but also the NF-**κ**B-IL-6-dependent pathway to some extent, thus leading to attenuate renal damage caused by the protein glycation reaction.

To test the direct anti-inflammatory action of FA, we measured its effect on the acute inflammation induced by carrageenan and dextran on rats. FA was found to exert anti-inflammatory effect which supports data obtained from the measurement of IL-6 in diabetic rats.

Controlling hyperglycemia in diabetic patients with insulin or other hypoglycemic agents and the reduction of oxidative stress, ROS production and inflammation result in the attenuation of diabetic complications especially DN. All of these findings support our hypothesis that FA has a renal protective role against oxidative damage, which may be due to its antioxidant/anti-inflammatory potential. Therefore, we accepted our hypothesis. In addition, metformin was selected as a positive control in the present study because of its well-known hypoglycemia effect. Table 1 shows that the hypoglycemic effect of all doses of FA was still lower than that of the positive drug MF. However, the high dose of FA reversed all of the renal lesions, inflammation, and oxidant/antioxidant status to almost the levels of the MF group. Therefore, our results might provide further insight into therapeutic strategies for diabetic kidney disease.

## Figures and Tables

**Table 1:** Physiological, biochemical, and renal functional parameters of the rats.

	Normal control	DN	DN + MF	DN + FA1	DN + FA2	DN + FA3
Initial body weight, g	190.3 ± 9.5	192.4 ± 8	191.5 ± 7.4	190.9 ± 7	193 ± 9.2	191.3 ± 6.9
Final body weight, g	265.5 ± 8.1	163.9 ± 9.2^a^	206.3 ± 8.5^a,b^	201.3 ± 17.5^a,b^	211.2 ± 8.9^a,b^	212 ± 11.3^a,b^
Glucose, mmol/L	4.98 ± 0.48	16.15 ± 1.55^a^	6.2 ± 1.59^a,b,d^	8.2 ± 1.5^a,b,d^	7.81 ± 1.3	7.31 ± 0.59^a,b,c^
Kidney/body weight, g/g, ×10^−3^	6.29 ± 0.35	11.2 ± 1.52^a^	8.1 ± 1.2^a,b^	8.5 ± 1.31^a,b,d^	7.9 ± 1.39^a,b^	7.5 ± 1.5^a,b,c^
BUN, mmol/L	6.81 ± 1.3	12.35 ± 3.1^a^	9.59 ± 1.65^a,b^	11.34 ± 1.35^a,d^	9.2 ± 1.1^a,b,c^	8.55 ± 1.29^a,b,c^
Serum creatinine, mmol/L	50.6 ± 7.1	65.12 ± 8.7^a^	57.9 ± 6.5^a^	62.1 ± 5.9^a,d^	54.3 ± 3.4^a,b,c^	52.16 ± 6.8^a,b,c^
Ccr mL min^−1^ kg^−1^	3.39 ± 0.4	6.39 ± 0.6^a^	4.52 ± 0.35^a,b^	4.45 ± 0.41^a,b^	3.98 ± 0.51^b^	3.75 ± 0.46^b,^
U prot, mg/24 h	8 ± 1.1	23.6 ± 2.5^a^	16.8 ± 2.5^a,b^	18.9 ± 2.1^a,b,d^	16.2 ± 2.9^a,b^	15.5 ± 2.25^a,b,c^
Serum sodium, mmol/L	145.1 ± 5.3	191 ± 10.5^a^	153.5 ± 13.7^a,b^	167.5 ± 8.9^a,b,d^	155.3 ± 9.3^a,b,c^	148.3 ± 2.3^b,c,d^
Serum potassium, mmol/L	4.77 ± 0.54	7.24 ± 0.98^a^	5.45 ± 0.5^b^	6.8 ± 0.51^a,d^	5.65 ± 0.66^a,b,c^	5.15 ± 0.54^b,c^
Serum IL-6, g/mL	81.2 ± 0.44	672.2 ± 15.6^a^	256.5 ± 13.2^a,b^	454.1 ± 1233^a,b,d^	344.8 ± 14.7^a,b,c^	261.2 ± 12.34^a,b,c,d^

Data are expressed as the means ± SD. DN: diabetes group; FA1, FA2, FA3, and MF: diabetic rats treated with low dose (5 mg/kg) of FA (DN + FA1), middle dose (50 mg/kg) of FA (DN + FA2), high dose (500 mg/kg) of FA (DN + FA3), and (125 mg/kg) of metformin (DN + MF), respectively. Each group consisted of 10 animals.

^a^ *P* < 0 .05 versus normal control group; ^b^ *P* < 0.05 versus DN group; ^c^ *P* < 0.05 versus DN + FA1 group; ^d^ *P* < 0.05 versus DN + FA2 group.

**Table 2:** Oxidant/antioxidant parameters as well as concentration of AGEs, CML, and IL-6 of rat kidney.

	Normal control	DN	DN + MF	DN + FA1	DN + FA2	DN + FA3
SOD, U/mg protein	21.3 ± 3.12	8.9 ± 2.1^a^	18.25 ± 1.55^a,b,d^	15.5 ± 1.8^a,b,d^	19.93 ± 1.4^b,c^	20.5 ± 1.55^b,c^
GSH, nmol/mg protein	25.6 ± 2.12	17.41 ± 2.13^a^	20.4 ± 2.2^a,b^	19.85 ± 2.19^a,b^	20.29 ± 2.17^a,b^	23.78 ± 1.33^b,c,d^
MDA, nmol/g protein	3.32 ± 0.2	7.1 ± 0.7^a^	5.1 ± 0.35^a,b,c^	6.2 ± 0.21^a,b,d^	5.05 ± 0.5	4.07 ± 0.13^b,c,d^
AGEs, AU	3.4 ± 0.2	7.1 ± 0.4^a^	4.2 ± 0.3^b,c^	6.2 ± 0.35^a,b,d^	4.85 ± 0.55^a,b,c^	3.9 ± 0.6^b,c^
CML, ng/mg protein	22.2 ± 5.5	48.16 ± 6.1^a^	30.5 ± 3.5^a,b,c^	40.8 ± 5.4^a,b,d^	32.3 ± 4.8^a,b,c^	29.5 ± 4.1^a,b,c,d^
IL-6, ng/mg protein	115 ± 2.5	1350 ± 45^a^	560 ± 24^a,b,c,d^	850 ± 35^a,b,d^	628 ± 31^a,b,c^	490 ± 28^a,b,c,d^

Data are expressed as the means ± SD. DN: diabetes group; FA1, FA2, FA3, and MF: diabetic rats treated with low dose (5 mg/kg) of FA (DN + FA1), middle dose (50 mg/kg) of FA (DN + FA2), high dose (500 mg/kg) of FA (DN + FA3), and (125 mg/kg) of metformin (DN + MF), respectively. Each group consisted of 10 animals.

^a^ *P* < 0  .05 versus normal control group, ^b^ *P* < 0  .05 versus DN group, ^c^ *P* < 0.05 versus DN + FA1 group, ^d^ *P* < 0  .05 versus DN + FA2 group.

**Table:** (a) Effect of FA on carrageenan-induced rat paw edema

Treatment	Thickness of the injected foot, mm	
	3 h	5 h
Olive oil	5.1 ± 0.12	5.03 ± 0.14
Indomethacin 10 mg/kg	1.68 ± 0.15 (62%)**	1.51 ± 0.10 (70%)**
FA, 5 ng/kg	3.16 ± 0.21 (38%)*	3.05 ± 0.09 (39%)*
FA, 50 ng/kg	2.95 ± 0.13 (42%)**	2.81 ± 0.17 (44%)**
FA, 500 ng/kg	2.18 ± 0.21 (57%)**	2.05 ± 0.15 (59%)**

**Table:** (b) Effect of FA on dextran-induced rat paw edema

Treatment	Thickness of the injected foot, mm	
	45 min	90 min
Olive oil	6.10 ± 0.17	5.90 ± 0.17
Indomethacin, 10 mg/kg	1.95 ± 0.15 (68%)**	1.66 ± 0.10 (72%)**
FA, 5 ng/kg	3.52 ± 0.16 (42%)*	3.1 ± 0.09 (47%)*
FA, 50 ng/kg	3.1 ± 0.21 (49%)**	2.75 ± 0.17 (53%)**
FA, 500 ng/kg	2.65 ± 0.12 (57%)**	2.25 ± 0.15 (62%)**

Values are mean ± SE, *n* = 6, *P < 0.05, **P < 0.001 compared with control, post-hoc test. Values given in parentheses represent percentage of inhibition.
